# Professional experiences with journal club implementation in postgraduate nursing: a qualitative study

**DOI:** 10.1186/s12912-025-03841-z

**Published:** 2025-09-10

**Authors:** Rasha A. Mohamed, Salwa Ali Marzouk, Mohammed Owayrif Alanazi, Nadia Falah Alenezi, Hadya Obied Alenzi, Naglaa Mustafa El-sayed, Rasha Fawzy Abdelmotaleb Gad, Nadia Ahmed El-Adham, Hanem Awad Mekhamier, Eman Shabaan Salah Hamd, Asmaa Abouda Abdelhameed Soultan, Wael G. Nouh, Asmaa Mohamed Ali AlAbd, Abeer A. Almowafy

**Affiliations:** 1 Community Health Nursing, Faculty of Nursing, Mansoura University, Mansoura, Egypt; 2 College of Applied Medical Sciences in Bisha, University of Bisha, P.O. Box 551, Bisha, Saudi Arabia; 3 Maternal and Child Health Department, College of Nursing, University of Hail, Hail, Saudi Arabia; 4 College of Nursing, University of Hafr Albatin, Hafr Albatin, Saudi Arabia; 5 Pediatric Nursing Department, Faculty of Nursing, Mansoura University, Mansoura, Egypt; 6 Family and Community Health Nursing, Faculty of Nursing, Damietta University, Damietta, Egypt; 7 Pediatric Nursing, Faculty of Nursing, Damietta University, Damietta, Egypt; 8 Medical Surgical Department, Faculty of Nursing, Damietta University, Damietta, Egypt; 9 Department of Psychiatric and Mental Health Nursing, College of Nursing, University of Hail, Hail, Saudi Arabia; 10 Department of Psychiatric and Mental Health Nursing, Faculty of Nursing, Benha University, Benha, Egypt; 11 International Islamic Center for Population Studies and Research, Al-Azhar University, Cairo, Egypt

**Keywords:** Journal club, Postgraduate nursing, Evidence-based practice, Critical appraisal

## Abstract

**Background:**

Postgraduate education is embracing journal clubs (JCs), which provide a platform for members to critically evaluate research articles and extract evidence-based nursing practice. The implementation of JCs by postgraduate nurses, especially in varied educational contexts such as Egypt, remains underexplored. This study aimed to explore and gain valuable insights into the professional experiences of implementing JCs among postgraduate nursing students in Egypt.

**Methods:**

A qualitative study utilizing focus group discussions (FGDs) was conducted with 42 postgraduate nursing students between September and December 2024. The participants were selected through purposive sampling. The inductive thematic analysis revealed several key themes.

**Results:**

The study identified five main themes: familiarity with JCs, benefits of JCs, preparation and implementation, challenges, and recommendations for enhancing the effectiveness of JCs. This study highlights the significance of JCs in promoting evidence-based nursing competencies, cultivating a research-oriented culture, and enhancing clinical application. Participants expressed concerns regarding time constraints and insufficient training, highlighting areas for improvement. It suggests integrating JCs into curriculum, aligning professional and academic responsibilities, and offering foundational research methodology training.

**Conclusions:**

The findings show that JCs are viewed by participants as worthwhile learning experiences that foster research literacy, academic development, and transferable skills including critical thinking, communication, and partnership. To enhance the effectiveness and sustainability of curriculum-integrated JCs, several strategies could be implemented: providing faculty training and introductory workshops in research appraisal; intentionally incorporating JCs into the nursing curriculum; allowing participants’ clinical and academic obligations; utilizing virtual platforms; and ensuring fair access to research materials. These observations, when combined, support the pedagogical worth of JCs as organized, practice-based learning resources that connect clinical education and research.

**Clinical trial number:**

Not applicable.

**Supplementary Information:**

The online version contains supplementary material available at 10.1186/s12912-025-03841-z.

## Background

Journal clubs (JCs) have long been recognized as effective educational tools for promoting evidence-based practice (EBP) and enhancing critical appraisal skills among healthcare professionals, including nurses [1–3]. Despite the acknowledged value of JCs in fostering EBP and critical thinking among nurses, there is a notable lack of qualitative research exploring their implementation and impact, particularly within postgraduate nursing education.

JCs are valuable tools for cultivating critical thinking, reasoning, and social skills. They can stimulate professional interest in medical and health fields while expanding local and international professional networks [4–6]. JCs provide a dynamic learning environment, bridge knowledge gaps, and serve as effective teaching tools for students, healthcare professionals, academic researchers, and future scientists [1, 7]. Additionally, they help students make informed decisions that lead to high-quality patient care [8–11].

Despite these advantages, JCs face several implementation challenges. These include organizational barriers, time constraints, and a lack of knowledge regarding research methodologies and statistics. Addressing these issues is essential for the successful implementation of JCs in postgraduate nursing education [1].

Although JCs are typically excluded from the undergraduate medical curriculum, they remain one of the most important components of postgraduate programs [12]. Today’s healthcare professionals must enhance their competencies to adapt to the evolving healthcare landscape. Staying informed about current research is essential to meet professional requirements, and this can be achieved through continuing education and training, which are further supported by JC sessions [13]. Therefore, postgraduate nursing students should actively engage in nursing research to apply published findings to clinical decision-making, enhance their critical evaluation skills for modern, effective treatment, and improve patient outcomes [14, 15].

Despite the potential benefits of journal clubs (JCs), there is a surprising lack of published information regarding their implementation and impact on clinical decision-making, professional development, and the application of evidence in practice, particularly in postgraduate nursing education [2, 16]. In-depth qualitative studies that explicitly examine the lived experiences, perceived advantages, challenges, and contextual factors influencing the establishment and sustainability of JCs among postgraduate nursing professionals are scarce. Most current studies focus on quantitative outcomes such as knowledge improvement or test scores [1]. Furthermore, the generalizability of previous research evaluating nurses’ perceptions of JCs is limited, as many studies have used self-structured questionnaires without standardized scales [17].

Given the substantial growth of nursing education in recent decades—evidenced by the increase in master’s programs in health professions education offered worldwide and the expanding body of related research—this gap in the literature is particularly noteworthy [2]. To the best of our knowledge, this is the first qualitative study to explore the experiences of postgraduate nursing students with journal club (JC) implementation in Egypt, addressing a significant gap in the literature. By focusing on how these experiences influence professional growth and evidence-based practice (EBP), this qualitative study helps fill the knowledge gap regarding the complex, lived experiences of postgraduate nurses with JC implementation.

The findings of this study will inform best practices for establishing JCs in this context by highlighting practical obstacles, such as time constraints and a lack of research training, and will contribute to the growing body of research on effective teaching strategies in postgraduate nursing education. Such studies provide valuable insights into the perceived benefits, challenges, and overall impact of JCs on developing research skills, critical appraisal abilities, and EBP in postgraduate nursing students. Incorporating JCs into postgraduate programs and addressing implementation challenges can make nursing education more dynamic, research-based, and adaptable to the evolving needs of the healthcare industry.

## Methods

### Study design

This study utilized a descriptive qualitative research design to provide in-depth insights into the experiences of postgraduate nursing students regarding the implementation of JCs in professional contexts. Merriam and Grenier (2019) [18] stated that qualitative research explores how individuals interpret their experiences and systematically construct their thoughts. The findings of this study were presented in accordance with the Consolidated Criteria for Reporting Qualitative Research (COREQ), which consists of a 32-item checklist for interviews and focus groups [19].

The primary method of data collection was FGDs, which allowed for the exploration of shared experiences among postgraduate nursing students. The recommended number of participants for FGDs ranged from six to eight, as noted in the literature [20]. Each of the six FGDs consisted of seven participants, chosen to ensure a variety of experiences, specialties, and practice settings. The FGDs were conducted in person, with participants required to have sufficient knowledge to engage in meaningful discussions on the topic. Purposive sampling was utilized to select participants to achieve this objective. Maximum variation sampling within this framework ensured diversity across clinical nursing specialties (e.g., community, medical-surgical, and critical care), educational backgrounds (master’s and postgraduate), age groups (< 30–40 years), and gender. This selection process aimed to identify information-rich cases to provide comprehensive insights into the implementation of JCs in nursing practice.

### Setting

This study was conducted in the Community Health Nursing Department at the Faculty of Nursing, Mansoura University, Egypt, between September and December 2024. The nursing department is a specialized academic and research center that was established in 1994. It provides a wide range of advanced courses and programs designed to enhance the knowledge and skills of nursing professionals, creating a comprehensive environment for postgraduate education. The department offers master’s and doctoral degrees in community health nursing as well as specialized master’s degrees in EBP and infection prevention and control.

### Participants and recruitment

Inclusion criteria for the study were as follows: (a) male and female postgraduate nursing students, (b) individuals who had presented at least once at a JC, (c) willingness to participate in FGDs, and (d) signing an informed consent form. Interns and those who expressed hesitation were excluded from the study. The participants’ diverse ages, genders, educational backgrounds, and varying years of schooling and work experience were highlighted. While all participants were postgraduate nursing students, some also had academic or professional obligations at work at the same time. As an illustration of role variety beyond merely student status, the category “Supervision on thesis/dissertation” includes participants who served as peer or junior student supervisors in clinical or academic contexts. In addition to completing their own postgraduate coursework, students may lead or mentor others in this dual position, which mirrors common professional growth trajectories.

Data saturation, indicating no new themes or insights emerging from further data collection, was used as a benchmark for determining the sample size [21]. Data collection and preliminary analysis were conducted concurrently. When more evidence supports the codes and themes found, but no new codes or themes can be found, researchers have reached inductive thematic saturation. After conducting six FGDs, inductive thematic saturation was reached regarding JC benefits, preparation and implementation, challenges, and recommendations for enhancing JC.

Data collection occurred over two months. Following similar qualitative studies, six FGDs were planned, each with seven participants (totaling 42). This number was deemed sufficient, as saturation was achieved after analyzing themes from all six sessions. Verbal invitations, assistance from the faculty academic advisor, and flyers were utilized for participant recruitment. Several precautions were implemented to minimize the risk of perceived coercion during student-faculty interactions. The faculty academic advisor’s sole responsibility was to provide access to the departmental email list; they were not involved in data analysis, FGD moderation, or contacting specific students. Invitation emails were sent by a research assistant who had no role in evaluating or supervising the students, and interested individuals contacted the assistant directly. FGDs were not attended by any teaching staff responsible for grading. Each FGD was facilitated by an impartial moderator who was not involved in evaluating the participants’ coursework. Students were informed verbally and in writing that declining or withdrawing from participation would not impact their academic or professional standing, and that participation was entirely voluntary. Prior to data analysis, all identifying information was removed during transcription and coding.

### Data collection

This study utilized a systematic and rigorous approach to collect data. Researchers carefully crafted a set of open-ended and follow-up questions to elucidate and explore similarities and differences in experiences during each FGD. An expert panel of qualitative researchers and nursing staff rigorously validated the guide. The review evaluated if the guide adequately covered the intended themes, ensured each question aligned with the study’s objectives, and confirmed that the questions were easy to understand, culturally appropriate, and free from ambiguity. The questions were translated from English to Arabic and vice versa to maintain consistency in meaning and consider the participants’ cultural backgrounds. Three students who were not part of the sample tested the Arabic version of the questions to assess the tool’s application, clarity, and reliability. Additionally, we aimed to determine the time needed for data collection. Data from this pilot study was not included in the final analysis.

Based on the findings of the pilot test, the questions were modified, and some statements were rewarded for clarity. The FGDs’ guide questions were formulated based on concepts from the literature to address professional experiences with journal club implementation in postgraduate nursing (Appendix 1). Two additional tools were used to enhance data collection: a field note template that recorded group dynamics, non-verbal cues, and emerging themes during sessions, and a demographic questionnaire that collected participant characteristics, such as age, sex, educational attainment, and previous exposure to JC-related educational sessions.

Initially, a list of students who had previously participated in JC was compiled from the postgraduate affairs office database. Subsequently, we invited all students to join by emailing them, outlining the study’s aim, and providing details about FGDs. The researchers explained the aims, procedures, risks, benefits, and confidentiality measures of the study to ensure comprehension. The participants signed a formal consent form before engaging in FGDs. All sessions were audio-recorded with participants’ permission to provide accurate data collection. The audio recordings were transcribed verbatim and reviewed for accuracy, with anonymization applied during the transcription. Following qualitative research guidelines, this method preserved the richness and complexity of the data. FGDs were conducted in a quiet, private faculty hall to minimize disruptions and distractions while accommodating participants’ academic schedules. Ground rules were established before the FGDs commenced, and a welcome greeting was provided to encourage effective communication. The researchers advised the participants to respect each other’s privacy, refrain from sharing information with third parties, and keep the identities of other FG participants confidential.

With seven participants, each FGD lasted roughly 30 to 45 min, resulting in relatively short speaking time for each participant. This structure allowed for in-depth discussion of the study’s core areas within a feasible time frame that was feasible for postgraduate schedules, although it limited the depth to which each participant could delve into every aspect of the subject. The six shorter sessions were effective, for example, by focusing on specific questions, repeating themes in FGD4, and collecting rich data from targeted prompts. However, we acknowledge that conducting additional interviews or longer sessions could have provided more detailed insights into specific subthemes.

To establish rapport, participants introduced themselves before the discussions began. The researchers encouraged the participants to express their emotions, anxieties, and concerns. In addition to sharing their thoughts, participants were strongly urged to respond to questions posed by the moderator and other participants. The researchers aimed to create a relaxed atmosphere that allowed participants to feel comfortable sharing their thoughts and opinions. They consciously avoided judgmental, dismissive, or negative comments and attitudes during FGDs. The questions progressed from general to specific, covering key domains. After the FGDs, the researchers employed follow-up queries such as “What else?” and “Is there anything else I should know beyond what I have asked?” Questions were used to clarify or expand on the participants’ responses.

The data did not reveal any new or significantly different topics, and the researchers repeatedly received similar responses from various participants, particularly when multiple responses overlapped. The data collector had no prior relationships with the participants. Five researchers were involved in the data collection process in a unique arrangement. All FGDs were moderated by N. M. with co-moderation provided by four other contributors: N. A., H. A., A. A., and E. S. Additionally, during the FGDs, the co-moderators took field notes, posed clarifying questions, and summarized discussions for the participants to review and expand upon.

### Research rigor

To ensure the trustworthiness of this study, we implemented strict criteria for credibility, transferability, dependability, and confirmability to mitigate potential bias [22]. Peer debriefing and member checking were employed to establish credibility and accurately interpret participants’ experiences. During the member check, researchers provided participants with their results to confirm accuracy and consistency with their experiences. A detailed explanation of the methods and procedures used in this study lends additional credence. In the results section, quotations are used to emphasize the conclusions. Two researchers (R. M. and A. A.) translated the quotations from Arabic to English. The manuscript proofreader, who had access to both the translated and original quotations, verified the accuracy of the English translation.

Transferability was ensured by providing a detailed account of the study history and confirming that the results were representative. A clear and comprehensive explanation of the procedures was presented to enable other reviewers to validate the findings. Confirmability was established through precise and timely documentation of emerging categories and subcategories. Additionally, the reliability of the results was enhanced by employing research triangulation in the analytical process.

Two researchers independently coded the transcripts to ensure rigor, and any discrepancies were resolved through discussion. By bracketing and setting aside personal biases, assumptions, ideas, and experiences, the researchers maintained a reflective stance to ensure that personal traits did not influence the research process. The researchers engaged participants for extended periods, communicated courteously and appropriately, and routinely reviewed the obtained codes with several individuals, while allowing ample time for data collection and processing. They indicated that their experiences were reflected in their identified topics.

Many authors of this study have previous experience with qualitative research. Each researcher possesses a wealth of research and teaching expertise, as well as training in qualitative research methods, analysis, and discussion. Most researchers hold PhDs, with 11 being female and three being male. Before collecting and processing the data, each researcher documented their prior knowledge to enhance awareness, intentionally applying it to foster a deeper understanding of the subject under investigation.

Researchers worked to create a respectable and open environment for the participants. Additionally, each finding was supported by verbatim examples from participants’ remarks. With the participants’ consent, the FGDs were recorded on two devices using the “Voice Memo” application on the phones of the two co-investigators.

In this study, an inductive thematic analysis was employed to categorize conversational text and identify trends for data analysis. After thoroughly reviewing the transcripts multiple times, we determined the codes and established connections between the ideas and codes. The coding process continued until data saturation was reached, which was achieved through iterative data collection, redundancy in findings, conceptual depth, and researcher reflexivity. A matrix or saturation table was used. The matrix included columns for both preidentified and emergent themes, with entries indicating whether a new concept appeared in a given FGD. The sixth FGD verified that all thematic domains were completely filled with recurring concepts, and by the fifth FGD, no new codes had been found. This demonstrated thematic saturation in areas such as JC benefits, preparation and implementation, challenges, and recommendations. As a traceable audit trail for how saturation was demonstrated, Supplementary Table S1 offers a simplified version of the saturation matrix that illustrates the development of code emergence and confirmation across FGDs. Saturation was confirmed through member checking and peer debriefing.

Inductive thematic analysis was conducted, following the six phases outlined by Braun and Clarke (2006) [23]. The process involved reading and noting initial codes, collating codes into potential themes. Additionally, transcripts were independently coded by two researchers to enrich reflexive engagement with the transcripts, and the coding methodology was refined through regular team meetings. In keeping with reflexive TA, no formal calculation of interrater reliability was performed, as coding was not aimed at measurement of rater agreement, but rather at generating nuanced and contextually grounded interpretations. Several vivo and descriptive codes were developed during the data analysis stage. Twenty-one distinct codes were identified in the six FGDs. Each code included a definition, its origin, and exemplary instances provided by participants. Regular meetings were held by coders to discuss and resolve disputes amicably, aiming to reach consensus rather than forced agreement.

After reviewing the information contained in these codes, informative memos were produced. These memos included a brief explanation of our analysis of the codes and selected examples from FGD transcripts. The memos outlined the themes discussed in the results section. Although the participants were not involved in the qualitative text analysis process, they were asked to review the themes to ensure an accurate representation of their experiences.

During the implementation of this study, emphasis was placed on aligning the study’s aim, research questions, design, data collection methods, and analysis to effectively address the research question. A reflexive approach was employed throughout the research process to improve the credibility and accountability of the study by allowing the participants to understand the researchers’ perspectives. Furthermore, researchers used reflexivity journals to record their prior knowledge, personal beliefs, evolving thoughts, and assumptions regarding the research topic throughout the analysis. We can identify instances where our positionality or past experiences impacted the application of codes by reviewing our reflexivity notes. This allows us to reexamine and modify the codes as necessary.

Reflexivity journals were shared to encourage discussion regarding varying interpretations and the influence of subjectivity on coding, which increased the rigor of the analysis. Representativeness were achieved by carefully selecting diverse FGD participants who provided extensive information to identify saturation levels. Furthermore, the research questions were developed by posing an exploration that explicitly seeks to understand what is known about a phenomenon from one or more perspectives, while considering the settings and environmental context.

### Data analysis

The Statistical Package for the Social Sciences (SPSS) was used to conduct a descriptive analysis of the participants’ personal and professional information. Inductive thematic analysis was performed to identify the key themes emerging from the FGDs. Following the principles set forth by Braun and Clarke (2006) [23], this analysis aimed to identify patterns, themes, and subthemes in the FGD transcripts. The inductive approach ensures that the findings are derived directly from the data, objectively capturing the nuances of participants’ experiences, particularly given the limited prior research on this topic. Two researchers independently coded the transcripts to enhance the transparency, trustworthiness, and replicability of the research findings. The final thematic analysis was developed through a collaborative process of refining the coding. Our reporting is descriptive and non-prevalence oriented.

## Results

The personal and professional characteristics of the participants are presented in Table 1.

**Table 1:** The distribution of personal and professional characteristics of the participants (*N* = 42)

Characteristics	*N* = 42	%
**Age (Years)**	x̄±SD (30.8 ± 6.4)	
< 30	22	52.4
30–40	11	26.2
> 40	9	21.4
**Gender**		
Male	25	59.5
Female	17	40.5
**Academic background**		
Master’s degree (MSN)	31	73.8
Postgraduate diploma/certificates	11	26.2
**Affiliation**		
Government hospital	22	52.4
Community Health	9	21.4
Academic setting	11	26.2
**Membership in professional associations** **job category**	24	57.1
Preparing for thesis/dissertation	8	19
Finishing thesis/dissertation	18	42.9
Preparing/working on a research paper	9	21.4
Supervision on thesis/dissertation	7	16.7
**Previous attendance of training sessions on research methods and critical appraisal**		
Yes	36	85.7
No	6	14.3

### Key themes identified

Thematic analysis of the FGDs identified five overarching themes and corresponding subthemes, which are summarized in Table 2.

**Table 2:** Themes and subthemes generated in this study

Themes	Subthemes
Familiarity with journal clubs	▪ Basic understanding of the journal club
Benefits of journal clubs	▪ Professional and academic skill development
	▪ Bridging the gap between academic learning and clinical practice
	▪ Achieving high-quality educational standards and accreditation
Preparation and implementation of the journal clubs	▪ Logistical support
	▪ Nomination of the JC’s champion
	▪ The role of mentors
	▪ Presentation of the journal club
Challenges that the participants faced during the journal club’s preparation and presentation	▪ Scheduling conflicts
	▪ Institutional limitations
	▪ Low attendance and inactive involvement
	▪ Disorganization in team dynamics
	▪ Difficulty in critical appraisal
	▪ Uneven skill distribution
Recommendations for improvement of the journal clubs	▪ Organizational structure
	▪ Flexible scheduling
	▪ Autonomy in article selection
	▪ Focus on practical relevance.
	▪ Shared educational backgrounds
	▪ Curriculum integration
	▪ Need for JCs’ training courses

### Theme 1: familiarity with journal club

#### Subtheme 1: basic Understanding of the journal club

Participants viewed JCs as a platform for discussing and criticizing research publications with instructors. A few participants expressed this view concisely, stating,

*I have only recently come across the term ‘journal club’. While I understand it’s a long-standing concept in academic circles*,* its widespread implementation seems more recent (FGD 6*,* P3).*

Although participants were familiar with the term, their interpretations of its meaning varied significantly, especially among those new to academic environments. They still have a basic understanding of journal clubs and how they function. These findings emphasize the need for more training and support to ensure that new members properly understand the objective and process of JC, thus maximizing their potential as valuable resources for professional development and research involvement.

### Theme 2: benefits of journal club

Participants identified the benefits of the JC to their professional work as a significant positive aspect.

#### Subtheme 2.1: professional and academic skill development

The role of JCs in enhancing participants’ exposure to research methodology, promoting skills in reading and critically reviewing research articles, and fostering scientific curiosity was emphasized by the participants. Some participants reflected on their experience, stating,

*Through our discussions*,* I’ve honed my ability to dissect research methodologies*,* interpret statistical analyses*,* and evaluate the real-world applicability of study findings (FGD 1*,* P4).*

Such observations are particularly significant in the context of postgraduate nursing education, where developing strong research skills is essential. A key insight is the students’ recommendation of JCs as venues for increased research to create a supportive community for aspiring researchers.

Furthermore, participants reported that JCs facilitate an in-depth literature review from a global nursing perspective, addressing the challenges nurses face and exploring potential solutions. They highlighted that JCs offer a structured environment to stay updated with the latest advancements in the nursing field. A few participants stated,

*JC serves as a starting point for conducting comprehensive literature reviews*,* analyzing our* difficulties, *and addressing care-related questions to resolve patient issues (FGD 2*,* P6).*

The experiences of the participants underscore how JCs support a culture of ongoing learning and professional development, enabling nurses to handle both everyday and emerging patient care issues.

Additionally, participants found the topics of the JC to be appropriate, engaging, and helpful for their knowledge and clinical needs. The JC assists participants in expanding their knowledge and viewpoints on specific topics related to nursing education, curriculum, and clinical issues. Several participants succinctly captured this perspective, stating,

*It provided new knowledge and perspectives that could be applied to my academic studies and clinical work (FGD 3*,* P1).*

This shows that the selection of content for the JC was well-suited to the needs and interests of the participants. This data suggests that JCs facilitate the transfer of knowledge and encourage the development of critical thinking and analytical skills.

What’s more, participants emphasized the role of JCs in providing opportunities to enhance communication skills, public speaking, and self-confidence. The JCs offered chances for participants to expand their networks by engaging with colleagues. Many participants highlighted these benefits, stating:

*The JC has helped me overcome initial public speaking discomfort*,* empowering me to express myself more effectively and assertively*,* a skill that will benefit me throughout my career (FGD 1*,* P2).*

This suggests that JCs serve as a platform for professional socialization, which is vital in healthcare settings. Professional socialization may lead to improved interdisciplinary collaboration in clinical practice, potentially increasing the sustainability of JCs. Furthermore, the diverse educational backgrounds of participants foster common understandings that enhance connections and partnerships at the bedside, while multidisciplinary sessions can facilitate richer and more valuable discussions.

Moreover, participants expressed the perception that JCs enhance academic performance, especially in courses like EBP, research projects, biostatistics, and epidemiology, which are prerequisites for nursing college. Participants also acknowledged that the effectiveness of JCs depends on comprehension and application rather than just memorization. Rarely, participants noted,

*JC’s skills have significantly enhanced academic performance across various disciplines*,* particularly in research projects*,* biostatistics*,* EBP classes*,* and epidemiology (FGD 4*,* P5).*

This understanding emphasizes that JCs are relevant across different nursing specialties and curricula. JCs can serve as an effective supplementary learning tool that reinforces and expands on formal coursework.

#### Subtheme 2.2: bridging the gap between academic learning and clinical practice

Participants stressed that JCs strengthen the connections between academic learning and clinical practice. They applied knowledge from journal articles to real-world experiences in hospitals and patient care techniques. Participants noted that, by applying evidence-based findings to real-world patient scenarios, JCs have the potential to deliver high-quality nursing care and improve decision making. This viewpoint was summed up by many participants, as they stated,

*It’s not just about accessing information; it’s also about developing critical skills to evaluate literature. Sharing scientific evidence can enhance the quality of care. (FGD 2*,* P4).*

These data indicate a strong perceived value among the participants. The high level of participant agreement suggests that JCs meet and fulfill critical needs in nursing practice and professional development.

#### Subtheme 2.3: achieving high-quality educational standards and accreditation

Participants indicated that engaging in JC activities can result in high-quality academic and professional accreditation in continuing nursing education. A few participants succinctly expressed this sentiment:

*JC activities have become essential for academic excellence in our faculty*,* setting a standard for education that continually motivates and challenges us to strive for greatness (FGD 6*,* P6).*

The participant’s statement emphasizes how JC activities can foster a culture of academic achievement. While individual participants benefit from this ongoing commitment to excellence, the reputation and effectiveness of the nursing faculty as a whole are also elevated.

### Theme 3: preparation and implementation of the journal club

#### Subtheme 3.1: logistical support

Participants emphasized specific organizational contexts for JCs to succeed. They highlighted the importance of scheduling the JC around essential clinical responsibilities and, if possible, relieving participants from clinical duties to attend. Ensuring reminders or follow-ups may be necessary before the event. Many participants shared this insight:

*At least two weeks prior to the meeting*,* I require information about the JC*,* including the date*,* time*,* location*,* subjects*,* and duration of each session. This is to ensure we can fully prepare and contribute meaningfully to the discussions (FGD 2*,* P3).*

Participants also noted that considering whether the JC will be held in person or virtually, with relatively brief sessions on alternate days, seems beneficial. Some participants explained:

*I prefer that JC meetings be relatively short in person or electronically on alternating days*,* especially for busy nurses (FGD 5*,* P4).*

The findings underscore how important organizational support and careful preparation are to the effective operation of JCs in healthcare settings and demonstrate an understanding of the diverse needs and preferences of participants.

Additionally, participants emphasized the importance of clear, specific, and time-bound goals for JCs. They also agreed that instructors should dedicate more time to outlining the learning objectives students are expected to achieve by participating in the activity. For instance, a few participants stated,

*Setting JC goals early on aids in creating a roadmap for discussions*,* influencing article selection*,* and focusing group efforts on achieving specific*,* measurable outcomes (FGD 5*,* P7).*

This deeper insight demonstrates that establishing clear goals can enhance participant motivation and engagement, potentially transforming the JC experience from a passive activity into an active, goal-oriented learning pursuit.

Furthermore, participants expressed the need to verbally introduce JC in class before starting the learning activity with written instructions that should be available on their digital learning platform. Additionally, they remarked that If JC’s goals had been clearly stated, they could have aligned their expectations and efforts better from the start. Some participants explained,

*A well-written introduction would have given me clarity and motivation to participate (FGD 4*,* P7).*

This finding highlights a strong preference for easily accessible information about JC procedures and expectations. Additionally, this preparatory step can help reduce confusion, create a positive environment, and ensure that everyone starts with a shared understanding of the activity’s purpose and importance.

#### Subtheme 3.2: nomination of the JC champion

A JC champion can serve as a motivator and role model, using peer influence to encourage participation and engagement, as reported by the participants. They stressed the importance of appointing a different presenter for each JC session to ensure that responsibilities are shared. Many participants shared:

*The champion should establish a visible session schedule*,* send timely reminders to participants*,* and ensure all members are aware of upcoming meetings (FGD 1*,* P5).*

By fostering a cooperative and encouraging learning environment where leadership and shared responsibility promote sustained involvement and academic performance, these tactics enhance the effectiveness of JCs.

#### Subtheme 3.3: the role of mentors

The importance of mentors in helping participants better understand the research materials was emphasized by the participants. They appreciated the exhaustive efforts their mentors put in to guide them in breaking down complex papers with intricate language and organization into simpler versions suitable for presentation. Several participants shared:

*At first*,* I struggled with the research paper’s complexity*,* feeling overwhelmed by its extensive material and technical terms. However*,* our mentor’s helpful guidance changed my experience (FGD 5*,* P1).*

The findings highlight that the mentors play a crucial role in increasing the educational value of JCs. They provide individualized guidance, remove obstacles to knowledge, and create a more welcoming and supportive learning environment for all participants.

#### Subtheme 3.4: presentation of journal club

The participants appreciated the opportunity to develop their presentation skills during the JC sessions. They demonstrated that the most common methods of presenting were individual reading, oral summaries of the study, and group discussions. The most effective way to introduce the session’s content was found to be through a PowerPoint presentation. Many participants summed up this perspective by stating,

*The most effective JC presentations involve concise visual aids*,* oral summaries*,* and group discussions*,* making it easier to understand complex research findings (FGD 2*,* P3).*

This response highlights the importance of incorporating structured visual presentations and discussion opportunities to enhance participants’ learning and engagement in JC sessions.

Furthermore, the participants reported that working in small groups to discuss and evaluate the article was valuable. Some participants explained,

*Working in small groups has great learning potential. It encourages active participation*,* allowing each member to voice their perspectives and contribute meaningfully to the work (FGD 3*,* P3).*

Participants gain knowledge from each other’s perspectives and experiences, which is particularly beneficial in a profession like nursing, where real-world application is essential. This method fosters group learning, critical thinking, and meaningful discussion.

### Theme 4: challenges that the participants faced during journal club Preparation and presentation

Although many participants acknowledged the value of JCs as a learning tool, they also believed there was a real chance of low attendance and inactive involvement. The participants cited many barriers.

#### Subtheme 4.1: scheduling conflicts

Participants expressed that attending JC was often challenging for them due to educational obligations such as lectures and exams, as well as professional work responsibilities. Many participants reflected on their experience, stating,

*Since members were dispersed throughout many institutions*,* in-person meetings were not always possible. In contrast*,* face-to-face interactions offer unique benefits (FGD 5*,* P1).*

Such statements illustrate that in-person meetings with rigid scheduling are not ideal for healthcare workers with varying shift patterns or those who live far away. These findings highlight the need for more flexible formats, such as virtual or hybrid JCs, to accommodate participants’ diverse schedules.

#### Subtheme 4.2: institutional limitations

According to the FGDs, participants noted a lack of support from the college, time, and JC instruction and training in some courses. Some students believed there wasn’t enough information about how to apply JCs in academic settings. For instance, some participants remarked,

*The diverse schedules*,* which include clinical practice*,* research commitments*,* and personal obligations*,* lead to conflicting availability (FGD 1*,* P4).*

The participant’s remark demonstrates that scheduling problems are a recurring obstacle, which is exacerbated by the lack of institutional support and direction. This leaves students without resources or clear frameworks to help them participate in JC. These findings highlight the importance of institutional support, a systematic approach, and flexible scheduling in improving JC effectiveness and participation.

#### Subtheme 4.3: low attendance and inactive involvement

The risk of low attendance and passive participation in JCs, where few members volunteer to present or participate actively, was acknowledged by the participants. A few participants expressed this concern:

*It isn’t very pleasant because JCs are meant to be about teamwork and idea exchange (FGD 1*,* P3).*

These patterns suggest that JCs may struggle to achieve their learning objectives without strategies to encourage broader participation, such as incentives, structured facilitation, or rotating presenter roles.

#### Subtheme 4.4: disorganization in team dynamics

Participants mentioned a variety of obstacles, such as interpersonal conflicts among teams and organizational issues. Many participants noted tension in how the team’s duties and meetings were conducted. Some participants expressed,

*With the meetings and the disputes within the team*,* I believe they could be a bit more organized (FGD 3*,* P4).*

This emphasizes the importance of implementing structured agendas, defined rules, and effective facilitation for JCs, which could help reduce conflicts and improve the overall functioning of the group.

#### Subtheme 4.5: difficulty in critical appraisal

Reviewing research articles was challenging for participants and a major learning obstacle, especially when it came to the critical evaluation component. This process was difficult because it required a higher degree of critical thinking, which can be abstract and sophisticated for those still honing these skills. Many participants expressed this difficulty with this aspect:

*It was a little difficult to understand the critical evaluation portion because it wasn’t like the other sections*,* where you just read and summarize them (FGD 3*,* P1).*

This illustrates the larger problem that critical evaluation is not always easy and often requires a certain amount of academic maturity and research process knowledge. It also highlights the importance of JC programs, including organized training on research appraisal.

#### Subtheme 4.6: uneven skill distribution

Participants demonstrated that some people felt secure and gave a good presentation, while others were anxious and self-conscious, especially when they compared themselves to more assured colleagues. A few participants expressed,

*I feel like I’m not as good as others who seem comfortable speaking in front of everyone (FGD 1*,* P7).*

This range of abilities highlights differences in the students’ past exposure or comfort levels with critical thinking and public speaking. These variations also emphasize the importance of providing a welcoming atmosphere where everyone is encouraged to participate, regardless of their initial level of confidence.

### Theme 5: recommendations for improvement of the journal club

Participants’ suggestions for improving JCs consider their ideas for making them more accessible, relevant, and well-structured.

#### Subtheme 5.1: organizational structure

A well-organized JC structure with clear roles and responsibilities was deemed essential for efficient operation and participant satisfaction, as pronounced by the participants. They stressed the importance of assigning clear leadership responsibilities, systematically selecting papers, facilitating discussions, and handling administrative work to ensure accountability and reduce the workload for each participant. These recommendations were summarized in some participants’ statements:

*When leadership is assigned and discussions are well-facilitated*,* we can focus on meaningful critical analysis rather than just going through the motions (FGD 1*,* P5).*

This suggests that by promoting active participation and accountability among members, a well-run JC not only enhances operational efficiency but also enriches the educational process.

#### Subtheme 5.2: flexible scheduling

Using online scheduling tools and asynchronous participation options, such as recorded sessions, to accommodate diverse schedules was recommended by the participants. They can catch up and participate at their own pace, even if they cannot attend in person.

*Staying involved is made much easier by the flexibility of recorded sessions and online scheduling tools (FGD 3*,* P6).*

This perspective highlights how digital tools can be strategically used in academic settings to improve engagement, meet deadlines, and overcome logistical obstacles.

#### Subtheme 5.3: need for jcs’ training courses

The participants emphasized the importance of training in research methods and the development of strong foundational research skills as essential prerequisites for JC activities. They recommended using predetermined questions when reviewing papers. Many participants noted,

*It would be impossible to evaluate studies without first understanding research methods (FGD 1*,* P2).*

This feedback allows for more meaningful discussions and assessments of research articles.

#### Subtheme 5.4: autonomy in Article selection

Participants shared that sustaining engagement can be achieved by offering members autonomy in article selection and incentives. The reading is more relevant and enjoyable. A few participants elaborated:

*I stay interested because I can select articles that fit my interests and line of work*,* especially when incentives are involved (FGD 3*,* P6).*

These findings suggest that offering incentives and allowing participants to choose articles are effective strategies for maintaining sustained interest and active involvement in JC activities. This also ensures that the topics are relevant to their interests and line of work, which increases intrinsic motivation.

#### Subtheme 5.5: focus on practical relevance

Participants recommend selecting recent, relevant, and engaging primary research or systematic reviews for future professional practice. They argue that JC sessions are most useful and meaningful when they focus on current research directly related to clinical practice. Some participants commented:

*Choosing recent primary research or systematic reviews that relate to our clinical practice makes JC discussions more meaningful (FGD 6*,* P4).*

This approach promotes evidence-based decision-making in professional settings by ensuring that discussions are both practically helpful and philosophically instructive.

#### Subtheme 5.6: shared educational backgrounds

Participants mentioned that having the same educational background helps in creating common understandings that enhance connections and partnerships at the bedside. Additionally, multidisciplinary sessions can lead to more enriching and valuable discussions. A few participants elaborated,

*Because members have similar knowledge bases*,* they can communicate and collaborate more effectively (FGD 5*,* P2).*

By leveraging the skills and experiences of various disciplines, these sessions are believed to provide greater, more valuable insights, ultimately improving clinical practice and patient care.

#### Subtheme 5.7: curriculum integration

The concern about incorporating JC into the curriculum was emphasized by the participants, especially in courses focusing on professional practice. JCs are considered highly relevant to courses that highlight professional practice and other real-world nursing and healthcare scenarios, as noted by the participants. Additionally, the participants emphasized the importance of aligning JC topics with case-based clinical scenarios and curriculum-relevant themes to avoid redundancy or irrelevance. Several participants shared,

*It would be very beneficial to incorporate JC into professional practice courses (FGD 5*,* P6).*

These findings suggest that for JCs to effectively teach nursing, their content must be carefully selected to reflect clinical practice realities and promote the development of evidence-based professional competencies.

## Discussion

The study investigates the professional experiences of postgraduate nursing students with JC implementation. It found that JC is valuable for academic and professional growth, as it enhances research skills, promotes creative thinking, improves reading comprehension, enables critical evaluation, and keeps students informed. Our findings suggest that the advantages of JC may be more pronounced at higher levels of professional education, possibly due to prior exposure to research training and more immediate clinical application opportunities, which align with previous studies. These studies advocate for the modernization of clinical procedures, the implementation of an EBP, and the enhancement of critical appraisal abilities [16, 17, 24–26].

Furthermore, these findings align with previous studies highlighting the importance of JCs in translating research into practical therapeutic insights [17, 27]. However, a study by Kim et al. (2020) [28] suggests that JCs may not consistently engage students or effectively motivate them to integrate EBP into their practice. This implies that the structure, facilitation, and integration of JCs into the curriculum can significantly impact their effectiveness. Additionally, another study [29] found that there is currently insufficient solid data in the literature about the long-term effects of JCs, and they might not result in better knowledge application or EBP. The literature lacks solid data on the long-term effects of JCs, their impact on knowledge application and evidence-based decision-making, as well as their influence on clinical behavior and patient outcomes [30]. This indicates the need for more comprehensive, outcome-focused research.

### Theme 1: familiarity with journal club

The study found that despite having advanced academic backgrounds, participants often misunderstand the term “JCs” due to curriculum gaps and contextual factors. It emphasizes the need for JCs to be incorporated into a coherent curriculum to ensure that goals are understood and active involvement is encouraged. According to Patil (2013) [31], JCs are occasionally not well incorporated into the overall curriculum, being handled more like stand-alone exercises than as a component of a cohesive research teaching strategy.

### Theme 2: benefits of journal club

Despite the purported advantages of JCs, systematic reviews have pointed out that there is not enough data to support the idea that JCs directly affect patient care outcomes and evidence-based nursing [1]. This study emphasizes the significance of JCs in facilitating professional development, encouraging productive clinical discussions, and implementing evidence-based practices, all within a comfortable and non-intimidating setting. Similar findings were also reported by Hassan et al. (2023) [17]. However, a study conducted by Samy et al. (2019) [32] discovered that for JCs to have a lasting impact on clinical practice, consistent follow-up and reinforcement are necessary, indicating that additional structural support may be beneficial. The study found that JCs facilitate the application of published articles and the formulation of evidence-based recommendations, thereby enhancing professional growth in nursing and allied disciplines. This data supports the findings of the Drayton study, which indicated that JC enhances participants’ professional experiences [33].

The study found that participating in JCs significantly improved participants’ understanding of research, bridging the gap between theoretical knowledge and practical application. This improvement was attributed to attending training sessions on research methodology and critical appraisal as well as having completed a bachelor’s degree in research and biostatistics. Similar findings were noted in a previous study [32]. Furthermore, a cross-sectional survey conducted in February 2019 in Sohar, Sultanate of Oman, yielded similar results [34]. The study found that JC sessions improved participants’ academic performance in research-related courses such as EBP, epidemiology, and biostatistics. However, self-selection among students who are already academically inclined may be a factor in the perceived benefits, suggesting that students who actively participate and perform well may benefit the most. The findings of our study were corroborated by previous research [35].

The current study emphasizes the importance of JCs in promoting education, self-directed learning, and professional growth. Our results contribute to the literature by offering a more detailed understanding of the processes that lead to these benefits. The study highlights the structured format of JCs, which involve breaking down complex research papers, and their role in fostering critical thinking and academic development. Our findings are consistent with previous studies [17, 35], which highlight the significance of JCs in promoting critical thinking, applying knowledge, and facilitating academic development.

The present study found that JCs offer advantages such as social networking, collaboration, and open discussion, involving all attendees. These findings are supported by reference [36]. Similarly, reference [37] noted that many participants engage more actively and productively in the JC discussions. However, another study contradicts our findings [38]. The study also revealed that uneven participation can limit the benefits of open discussion. It suggests the need for intentional facilitation techniques to ensure balanced participation, challenging the notion of JCs being naturally inclusive.

### Theme 3: Preparation and implementation of the journal club

Participants preferred online journal clubs over traditional in-person ones due to their familiarity with digital platforms, efficient use of time, open discussion, and revisitability. This trend is influenced by digital literacy and adaptable learning settings. These results align with those reported in previous studies [38–41]. However, a qualitative survey by Al-Imari et al. (2020) [42] presents a notable counterpoint, as it found that medical students and residents preferred small-group, face-to-face journal club formats over virtual ones. More research is needed to determine the optimal online-to-in-person ratio.

Mentors are essential for the success of a JC (Job Coach) by providing support, guidance, and encouraging innovation [43]. Participants in the current study emphasized the importance of creating a secure, relaxed, and supportive environment facilitated by the mentor. They noted that using emoticons for positive feedback during presentations, providing guidance during practice sessions, and simplifying complex research concepts were particularly beneficial. This study supports the notion of mentorship’s benefits and emphasizes cutting-edge approaches such as psychological safety and digital feedback. These findings are consistent with a survey conducted by Ranganath (2019) [12] on JCs in family practice residencies in the United States, which revealed that the effectiveness of JCs is strongly linked to having a single, dedicated leader.

### Theme 4: challenges that the participants faced during journal club preparation and presentation

Despite widespread support for nursing journal clubs’ educational and professional benefits, conflicting evidence about their true effects on clinical practice and patient care still exists [1]. This study found that nursing journal clubs can improve critical appraisal skills, but some participants struggled due to a lack of necessary information and understanding of research methodology. Similarly, participants from Alfaisal University encountered difficulties understanding complex research papers due to a lack of foundational knowledge in research [5]. In contrast to the study’s conclusions, another study [24] found that regular participation in JCs can significantly enhance critical evaluation skills. The current study suggests that challenges in critical appraisal can be overcome with regular participation, structured guidance, flexible formats, and appropriate educational support.

### Theme 5: recommendations for improvement of the journal club

Choosing members with similar clinical interests or disciplinary backgrounds, establishing goals specific to articles, developing appraisal skills, and selecting case-based articles that are relevant to everyone are some of the integrated strategies that our participants identified for increasing JC engagement. Together, these components increase participation and investment by directly connecting discussions to everyday practice. Meeting a variety of learning demands and encouraging collaboration and career advancement required modifying JC activities with customized objectives and interactive forms. On the other hand, disengagement resulted from passive lecture formats. These results highlight the need for adaptable, participant-centered design and support Farrington’s (2022) [3] assessment of JCs as potent tools for applying evidence.

The importance of learning research skills before or during JC activities was emphasized by the study participants. To encourage consistency and thoroughness in critical assessment, they promoted the use of a predetermined set of structured questions during article reviews. According to these suggestions, adding research skills-focused workshops, seminars, or online modules to JC programs could give participants—especially those with little to no prior research experience—the necessary tools. Our study findings are consistent with those of previous research [44]. By highlighting the need for structured evaluative procedures and preceding instruction to optimize JC impact, especially for novices, this study adds to the body of knowledge.

There is much debate in EBP pedagogy regarding whether JCs by themselves are sufficient to convert research into practice. Comprehensive evaluations reveal that JCs improve reading habits and confidence in critical evaluation, although it is still unclear how they directly affect clinical conduct and patient outcomes. Better academic performance and research understanding were reported by study participants; however, these findings may be the consequence of intrinsic academic desire and tendency rather than the intrinsic efficacy of JCs. The report also highlights that without clear objectives, curriculum integration, and basic research training, JCs risk becoming isolated initiatives with limited potential for change. Using asynchronous online forums and open-access journals, developing leadership succession plans, and training new facilitators to keep things going when staff or students change are all practical ways to maintain journal clubs in a variety of educational contexts. In order to adapt JCs to particular learning contexts and participant demands, nursing educators must assess how well they integrate them into curricula. Furthermore, it is essential to conduct follow-up studies to assess the long-term impacts of JC engagement on clinical judgment and the adoption of EBP.

### Implications for research and practice

Educators, institutions, and researchers have an opportunity to significantly enhance the effectiveness of nursing journal clubs in promoting EBP and improving patient care outcomes. Based on the challenges outlined in Theme 4 and the recommendations in Theme 5, a structured approach is essential. For nursing educators, developing tailored journal club training programs responsive to the needs of different nursing specialties—while accounting for ethical considerations and research skill levels—can foster deeper engagement. Employing critical evaluation frameworks such as CASP will guide members through systematic assessment and meaningful discussion, moving beyond logistical concerns toward true critique. Integrating journal clubs into nursing curricula helps bridge the divide between theoretical knowledge and clinical application, reinforcing the practical relevance of research for students. Flexible scheduling and motivational strategies further support learning outcomes and increase engagement. Incorporating reflective exercises, such as written reflections or group debriefs after each session, strengthens the connection between evidence and clinical experience, enabling nurses to identify areas for professional growth. Peer-led discussions, mentorship, and small group activities build collaborative and critical appraisal skills, while the use of virtual platforms expands participation and accessibility for both busy and remote learners. These targeted strategies collectively create a robust framework for sustaining high-impact journal clubs that drive both educational and clinical excellence.

For institutions, enhancing the impact of nursing journal clubs involves establishing journal club participation as an institutional expectation and incentivizing involvement by recognizing it as part of professional development credits or continuing education. Encouraging participation from allied health professionals and other departments can foster interprofessional learning and broaden perspectives, enriching the overall discussion and collaborative potential of journal clubs. Promoting sustainability requires developing leadership transition plans and providing training for facilitators to ensure continuity and long-term success. Organizational support is critical and should include access to research resources and the allocation of dedicated time for journal club participation. Additionally, appointing committed journal club champions or leaders responsible for coordinating meetings and maintaining momentum helps sustain engagement and drive the ongoing effectiveness of these educational initiatives.

For future researchers, several avenues merit exploration to deepen and extend understanding of nursing JCs. Investigating the effectiveness of different mentorship models could best support participants’ research skills and overall JC outcomes. Comprehensive studies, including controlled trials and mixed-methods research, are needed to evaluate the long-term impact of JCs on clinical practice and patient care, as current evidence remains limited. Researchers should also examine the extent to which JCs influence the actual adoption of evidence-based nursing practices, addressing an existing gap in robust outcome data. Cross-cultural comparative studies could illuminate how varying organizational cultures and healthcare systems affect JC implementation and success, helping to tailor approaches across diverse contexts. Additionally, exploring how different JC formats—such as content-focused versus critical evaluation—and delivery methods (in-person versus virtual) impact participant learning, engagement, and knowledge translation will inform best practices for maximizing educational and clinical effectiveness.

### Strengths and limitations

One of the strengths of this study lies in its ability to capture the lived experiences of postgraduate nursing students as they reflected on the use of JCs in their academic and clinical contexts. Alongside findings from related studies, these results may contribute to building a broader understanding of how JCs can be applied in practice and inform the design of programs aimed at supporting evidence-based learning in nursing education. The focus group methodology enabled an in-depth exploration of participants’ perspectives while adhering to systematic analysis procedures.

However, a number of restrictions must be mentioned. First, since students who were more motivated or had previously shown an interest in the research were more likely to participate, the results may have been impacted by self-selection bias. Second, the qualitative design limits the results’ applicability to other professional groups or educational contexts by prioritizing depth over breadth. Third, the results are context-specific since the study’s geographic focus on Mansoura, Egypt, reflects institutional and cultural traits that might be different from those in other areas or nations.

There is also a chance that the researchers’ professional experience with JC education influenced study design, data collecting, or interpretation, which could lead to researcher bias. Even though reflexivity and methodical analysis were used to reduce this influence, it should still be considered when interpreting the findings. Finally, the study used focus groups, which could limit the range of opinions voiced due to group dynamics like social desirability or dominant voices. The study’s findings should be viewed as exploratory insights rather than final conclusions because of these constraints, providing initial direction that can be tested and expanded upon in larger contexts in future research.

## Conclusion

This study investigates the ways that JCs can foster a culture that is focused on research, assist in the clinical implementation of research findings, and possibly advance evidence-based nursing competency. According to participants, JCs may promote teamwork, critical thinking, and evidence-based decision-making. Inadequate training and time restrictions were also noted as obstacles to effective participation. The results imply that these problems could be resolved by providing basic training in research methods and by structuring JCs to better fit academic and professional obligations. According to the study, JC structures and processes can contribute to professional growth, and resolving logistical issues in addition to skill development may improve their ability to connect clinical practice and research. When tailored to local needs and resources, a curriculum-integrated journal club seemed to be beneficial in fostering postgraduate nursing students’ research literacy, critical thinking, and collaborative learning in the Egyptian environment examined here. These conclusions, however, are based on single department research conducted at a particular university, and variables including institutional scheduling procedures, national training needs, and resource availability vary depending on the environment. Therefore, rather than being proof that comparable results can be anticipated everywhere, the findings should be viewed as an analytical generalization that identifies mechanisms and tactics that may be helpful in different situations. Carefully incorporating JCs into postgraduate nursing programs could help develop a new generation of nurses who are not only knowledgeable about research but also self-assured team players and lifelong learners. Additional research could look at how these mechanisms function in various institutional and cultural contexts.

## Supplementary Information

Below is the link to the electronic supplementary material.

Supplementary Material 1

Supplementary Material 2

## Data Availability

Data is provided within the manuscript or supplementary information files.
