# Supplementary material for: Professional experiences with journal club implementation in postgraduate nursing: a qualitative study
Source: BMC Nurs. 2025 Sep 10;24:1166. doi: 10.1186/s12912-025-03841-z (PMC12424205; doi:10.1186/s12912-025-03841-z)
Supplement: Supplementary file 1 — Supplementary Material 1 [file 12912_2025_3841_MOESM1_ESM.docx]

**Appendix 1. The interview questions for the professional experience of postgraduate nurses with the journal club implementation**

**Introduction**

“Thank you for agreeing to participate in this interview. I’m interested in learning about your experiences as a postgraduate nurse participating in or implementing a journal club. There are no right or wrong answers—we're interested in your honest thoughts and reflections. This conversation will remain confidential. With your permission, I will record the interview to ensure accuracy in capturing your insights.”

| - How would you define a JC based on your professional experience? - What benefits or advantages do you believe JCs offer to postgraduate nurses? - How do you typically prepare for a journal club session? - What strategies have you found effective in encouraging participation and engagement during JC meetings? - What difficulties or challenges does the implementation of a JC present? - How have these challenges been addressed, if at all? - What recommendations would you suggest for enhancing the effectiveness of JCs? - Would you please tell me more about any memorable experiences or lessons learned from your involvement with JCs? - How could the journal club better support the professional development of postgraduate nurses? - Is there anything else you would like to add regarding the role or impact of JCs in your professional development? |
| --- |
