# Supplementary material for: Professional experiences with journal club implementation in postgraduate nursing: a qualitative study
Source: BMC Nurs. 2025 Sep 10;24:1166. doi: 10.1186/s12912-025-03841-z (PMC12424205; doi:10.1186/s12912-025-03841-z)
Supplement: Supplementary file 2 — Supplementary Material 2 [file 12912_2025_3841_MOESM2_ESM.docx]

**Supplementary Table S1 – Saturation Matrix showing theme emergence across Focus Group Discussions**

| **Theme / Main Code** | **FGD1** | **FGD2** | **FGD3** | **FGD4** | **FGD5** | **FGD6** |
| --- | --- | --- | --- | --- | --- | --- |
| **Familiarity with journal clubs** | ✓ | ✓ | ✓ | ✓ | ✓ | ✓ |
| **Benefits of journal clubs** – Bridging academia & practice | ✓ | ✓ | ✓ | ✓ | ✓ | ✓ |
| Benefits – Research capability development | ✓ | ✓ | ✓ | ✓ | ✓ | ✓ |
| Benefits – Navigating nursing literature | ✓ | ✓ | ✓ | ✓ | ✓ | ✓ |
| Benefits – Expanding knowledge & perspectives | — | ✓(N) | ✓ | ✓ | ✓ | ✓ |
| Benefits – Building networks, communication, teamwork | ✓ | ✓ | ✓ | ✓ | ✓ | ✓ |
| Benefits – Enhancing academic performance | ✓ | ✓ | ✓ | ✓ | ✓ | ✓ |
| Benefits – Achieving educational standards / accreditation | — | — | ✓(N) | ✓ | ✓ | ✓ |
| **Preparation & implementation** – Logistics | ✓ | ✓ | ✓ | ✓ | ✓ | ✓ |
| Preparation – Goal/Objectives setting | ✓ | ✓ | ✓ | ✓ | ✓ | ✓ |
| Preparation – Introductory JC session | ✓ | ✓ | ✓ | ✓ | ✓ | ✓ |
| Preparation – JC Champion nomination | — | ✓(N) | ✓ | ✓ | ✓ | ✓ |
| Preparation – Role of mentors | ✓ | ✓ | ✓ | ✓ | ✓ | ✓ |
| Preparation – Value of small group work | ✓ | ✓ | ✓ | ✓ | ✓ | ✓ |
| Preparation – Presentation delivery | ✓ | ✓ | ✓ | ✓ | ✓ | ✓ |
| **Challenges** – Scheduling conflicts | ✓ | ✓ | ✓ | ✓ | ✓ | ✓ |
| Challenges – Institutional limitations | ✓ | ✓ | ✓ | ✓ | ✓ | ✓ |
| Challenges – Low attendance / inactive involvement | ✓ | ✓ | ✓ | ✓ | ✓ | ✓ |
| Challenges – Disorganization in team dynamics | — | ✓(N) | ✓ | ✓ | ✓ | ✓ |
| Challenges – Difficulty in critical appraisal | ✓ | ✓ | ✓ | ✓ | ✓ | ✓ |
| Challenges – Uneven skill distribution | — | — | ✓(N) | ✓ | ✓ | ✓ |
| **Recommendations** – Organizational structure | ✓ | ✓ | ✓ | ✓ | ✓ | ✓ |
| Recommendations – Flexible scheduling | ✓ | ✓ | ✓ | ✓ | ✓ | ✓ |
| Recommendations – Autonomy in article selection | ✓ | ✓ | ✓ | ✓ | ✓ | ✓ |
| Recommendations – Focus on practical relevance | ✓ | ✓ | ✓ | ✓ | ✓ | ✓ |
| Recommendations – Shared educational background | — | ✓(N) | ✓ | ✓ | ✓ | ✓ |
| Recommendations – Curriculum integration | ✓ | ✓ | ✓ | ✓ | ✓ | ✓ |
| Recommendations – JC training courses needed | — | — | ✓(N) | ✓ | ✓ | ✓ |
| Number of new themes emerging in FGD | 6 | 4 | 3 | 0 | 0 | 0 |

**✓** = Theme present in discussion **—** = Theme absent in that FGD **(N)** = Theme newly emerging in that FGD

“New themes emerging” row shows how many *new* themes appeared at each stage; zero after FGD4 indicates saturation.
